# Dynamic gating of perceptual flexibility by non-classically responsive cortical neurons

**DOI:** 10.21203/rs.3.rs-4650869/v1

**Published:** 2024-07-23

**Authors:** Jade Toth, Blake Sidleck, Olivia Lombardi, Tiange Hou, Abraham Eldo, Madelyn Kerlin, Xiangjian Zeng, Danyall Saeed, Priya Agarwal, Dylan Leonard, Luz Andrino, Tal Inbar, Michael Malina, Michele N. Insanally

**Affiliations:** 1 Department of Otolaryngology, University of Pittsburgh School of Medicine, Pittsburgh, PA 15213; 2 Department of Neurobiology, University of Pittsburgh School of Medicine, Pittsburgh, PA 15213; 3 Department of Bioengineering, University of Pittsburgh, Pittsburgh, PA 15213; 4 Pittsburgh Hearing Research Center, University of Pittsburgh, Pittsburgh, PA 15213; 5 Center for the Neural Basis of Cognition, Carnegie Mellon University, Pittsburgh, PA 15213

## Abstract

The ability to flexibly respond to sensory cues in dynamic environments is essential to adaptive auditory-guided behaviors. Cortical spiking responses during behavior are highly diverse, ranging from reliable trial-averaged responses to seemingly random firing patterns. While the reliable responses of ‘classically responsive’ cells have been extensively studied for decades, the contribution of irregular spiking ‘non-classically responsive’ cells to behavior has remained underexplored despite their prevalence. Here, we show that flexible auditory behavior results from interactions between local auditory cortical circuits comprised of heterogeneous responses and inputs from secondary motor cortex. Strikingly, non-classically responsive neurons in auditory cortex were preferentially recruited during learning, specifically during rapid learning phases when the greatest gains in behavioral performance occur. Population-level decoding revealed that during rapid learning mixed ensembles comprised of both classically and non-classically responsive cells encode significantly more task information than homogenous ensembles of either type and emerge as a functional unit critical for learning. Optogenetically silencing inputs from secondary motor cortex selectively modulated non-classically responsive cells in the auditory cortex and impaired reversal learning by preventing the remapping of a previously learned stimulus-reward association. Top-down inputs orchestrated highly correlated non-classically responsive ensembles in sensory cortex providing a unique task-relevant manifold for learning. Thus, non-classically responsive cells in sensory cortex are preferentially recruited by top-down inputs to enable neural and behavioral flexibility.

## Introduction

All living organisms are required to flexibly navigate their environment for survival. For example, selecting the appropriate response to the sound of rustling leaves can have significant consequences for all animals, helping it find a mate or flee an approaching predator. Determining the appropriate response when faced with the same sensory cues often requires animals to suppress a previously learned sensorimotor action in favor of a new one (i.e. learning requires unlearning). Learning to adapt under changing task demands is believed to require the coordination of several brain areas including sensory and frontal cortex yet this process remains poorly understood given the vast heterogeneity in neural responses often observed in behaving animals^1–5^. Neural responses to sensory input during behavior are diverse, ranging from highly-reliable ‘classical’ responses (i.e. robust, frequency-tuned cells) to irregular or seemingly random ‘non-classically responsive’ firing patterns (i.e. nominally non-responsive cells) that fail to demonstrate any significant trial-averaged responses to sensory inputs or other behavioral factors^2–4,6,7^. This response heterogeneity is found across many brain regions ranging from visual cortex^8–12^, auditory cortex^2–4,6,7,13–17^, somatosensory cortex^18,19^, parietal cortex^5,11^, frontal cortex^4,20–22^ to subcortical areas such as the hippocampus^17,23–25^, hypothalamus^26^, and the ventral tegmentum^27^. Classically-responsive neurons are often referred to as tuned, selective, or responsive while non-classically responsive neurons are also described as untuned, non-selective or non-responsive^3,4,12,19,28,29^.

While classically responsive cells have been extensively studied for decades, the contribution of non-classically responsive cells to behavior has remained underexplored despite their prevalence. Previously, we showed that non-classically responsive cells in auditory and frontal cortex not only contain significant information about sensory stimuli and behavioral decisions in their spike times but also encode flexible task rules^4^. Neurons in monkey prefrontal cortex that are non-selective for working memory have been shown to improve population-level decoding on working memory tasks^29^. Single-unit recordings from human hippocampus has shown that in addition to strongly-tuned cells, weakly-tuned cells which comprised the majority of recorded cells, are important for encoding sequential memories^17^. In deep learning networks where the tuning of units was adjusted, increasing unit selectivity impaired task performance while decreasing it improved performance^30^. These findings suggest that non-classically responsive cells are beneficial for encoding a wide range of perceptual and cognitive variables spanning multiple species and brain areas. Recently, we showed in a spiking recurrent network model that non-classically responsive units are essential for asymptotic task performance^3^, however their role during learning *in vivo* is unknown. Here, we take advantage of an auditory reversal learning task in mice to examine how diverse cortical responses (including all recorded cells irrespective of their response profile) emerge and evolve during flexible behavior, and how long-range top-down inputs from frontal cortex shape these responses to alter circuit computations for enabling flexible behavior and learning.

## Results

### Auditory reversal learning depends on auditory cortex

**Figure 1.**
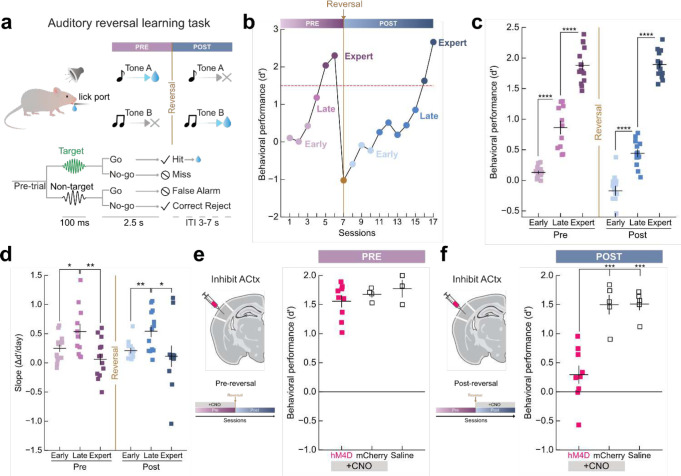
Auditory reversal learning is dependent on auditory cortex. **a**, Top, schematic of go/no-go auditory reversal learning task. Bottom, detailed task structure with behavioral outcomes. **b**, Example learning curve identifying three key learning phases during both pre-and-post-reversal: ‘early’ learning when animals performed near chance (< 40% progress towards max d’); ‘late’ learning when behavioral performance rapidly improves (≥ 40% progress towards max d’); and ‘expert’ performance (d’ ≥ 1.5 and percent correct ≥ 70%). **c**, Behavioral performance across learning phases. Pre_early_ (d’= 0.13±0.08, N = 15 mice) vs. Pre_late_ (d’= 0.86±0.33, N = 11 mice), p = 6×10^−8^; Pre_late_ vs. Pre_expert_ (d’= 1.8±0.27, N = 15 mice), p = 4×10^−8^; Post_early_ (d’= −0.13±0.20, N = 13 mice) vs. Post_late_ (d’= 0.46±0.18, N = 13 mice), p = 1×10^−6^; Post_late_ vs. Post_expert_ (d’= 1.8±0.21, N = 14 mice), p = 4×10^−15^. **d,** Learning curve slopes for each phase for animals in **c**. Pre_early_ (slope=0.25±0.21) vs. Pre_late_ (slope=0.54±0.41), p = 0.03; Pre_late_ vs. Pre_expert_ (slope=0.06±0.31), p = 9×10^−3^; Post_early_ (slope=0.21±0.15) vs. Post_late_ (slope=0.54±0.34), p = 9×10^−3^; Post_late_ vs. Post_expert_ (slope=0.11±0.56), p = 0.03. **e**, Bilateral chemogenetic inactivation of ACtx during pre-reversal. hM4D (d’=1.5±0.29, N = 9 mice) vs. mCherry (d’=1.6±0.10, N = 4 mice), p = 0.4 and saline control (d’=1.7±0.20, N = 3 mice), p = 0.4. **f**, Behavioral performance during post-reversal. hM4D (d’=0.29±0.43, N = 9 mice) vs. mCherry (d’=1.5±0.32, N = 5), p = 2×10^−4^ and saline control (d’=1.5±0.21, N = 5), p = 2×10^−4^. Data are mean+s.e.m. *p<0.05, **p<0.01, ***p<0.001, ****p<0.0001; two-sided independent t-test with Benjamini-Hochberg correction.

To determine how auditory cortical circuits are modified during learning, we trained mice on a go/no-go auditory reversal learning paradigm (Fig. 1a). Mice were first trained to respond to a target tone (11.2 kHz) by licking for a water reward, and to withhold their response to a single non-target tone (5.6 kHz), this stage is referred to as ‘pre-reversal’ (abbreviated ‘pre’). Once mice reached behavioral criteria (d’: ≥1.5 and percent correct ≥70%), we reversed which tone was rewarded by ceasing to reward the 11.2kHz tone and rewarding a previously non-rewarded tone (5.6 kHz), referred to as ‘post-reversal’ (abbreviated ‘post’). This paradigm required animals to relearn the task with different reward contingencies while ensuring they have learned the core task structure. Mice learned this task within a few weeks performing at high d’ values (Fig. 1b,c; Post_expert_ d’ = 1.8±0.2; **Extended Data Fig. 1a,b**). We identified three key learning phases: (1) ‘early’ learning when animals performed near chance (< 40% progress towards max d’ for a given animal) (2) ‘late’ learning when behavioral performance rapidly improves (≥ 40% progress towards max d’) and (3) ‘expert’ performance (d’ ≥ 1.5 and percent correct ≥ 70%) (Fig. 1b,c; **Extended Data Fig.1b-d**). These key learning phases were observed during both pre and post reversal and captured distinct learning dynamics (Fig. 1c, N = 15 mice). Examining the slope of all individual learning curves revealed that the greatest gains in behavioral performance occurred during ‘late’ learning (during both pre-and-post reversal) when performance improved rapidly, identifying a unique period of learning (Fig. 1d, Pre_late vs._ Pre_early_, p = 0.03; vs. Pre_expert_, p = 9×10^−3^; Post_late_ vs. Post_early_, p = 9×10^−3^; vs. Post_expert_, p = 0.03). To determine whether reversal learning depends on auditory cortex (ACtx) we performed bilateral chemogenetic inactivation experiments during pre-reversal or post-reversal training by expressing inhibitory DREADDs (hM4Di) in excitatory neurons (Fig. 1e,f; **Extended Data Fig. 1e**). Treatment of mice with CNO, which activates the inhibitory hM4D(Gi) receptor significantly impaired learning during post-reversal (Fig. 1f; after CNO injection, hM4Di: d’=0.3±0.4 vs. mCherry control: d’= 1.5±0.3, p = 2×10^−4^; after saline injection, d’= 1.5±0.2, p = 2×10^−4^) but not pre-reversal (Fig. 1e; after CNO injection, hM4Di: d’= 1.5±0.3 vs. mCherry control: d’= 1.6±0.1, p = 0.4; after saline injection, d’= 1.7±0.2, p = 0.4). Notably, general motor function, motivation, and ability to lick was not impaired following inactivation (p = 0.2; **Extended Data Fig. 1f)**. These results demonstrate that the ability to remap stimulus-reward contingencies depends on ACtx, but initial task acquisition does not.

### Diverse cortical responses measured during reversal learning

**Figure 2.**
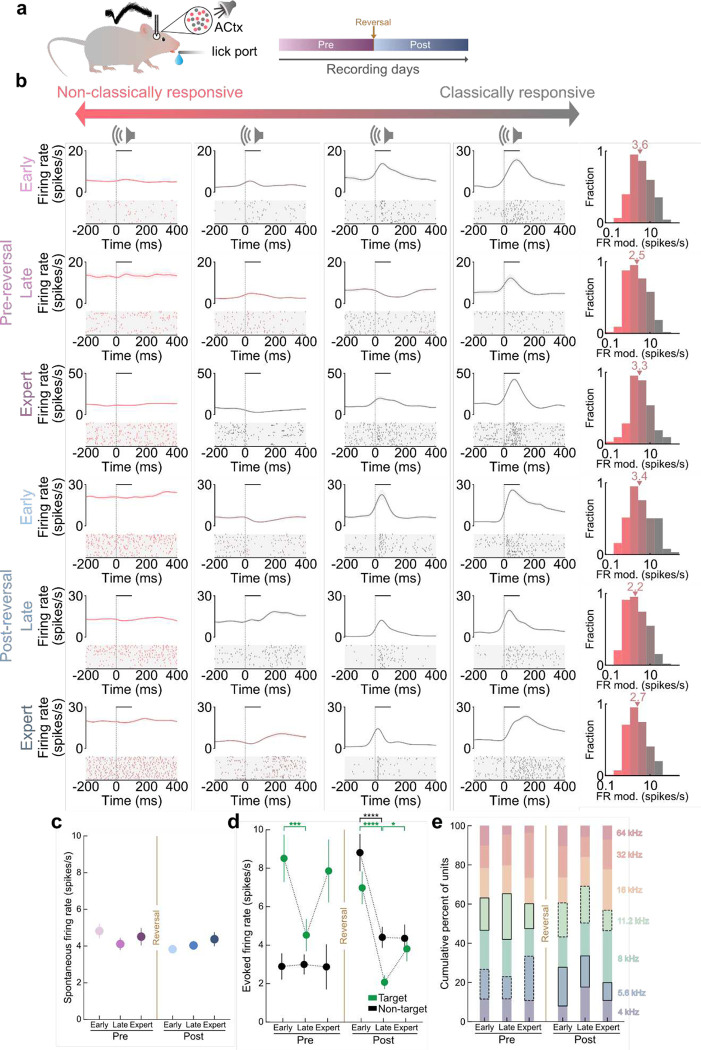
Heterogeneous cortical responses recorded from mouse auditory cortex during reversal learning. **a**, Schematic of extracellular single-unit recordings from mice performing a reversal learning task. **b**, Left, example peri-stimulus time histograms (PSTHs) and spike rasters from twenty-four cortical neurons exemplifying the range of stimulus responses from non-classically responsive (red, NCR) to classically responsive (gray, CR) during all stages of reversal learning. Lines in PSTH, mean firing rate; shading, s.e.m. Horizontal bar, tone duration. A subset of trials is shown in rasters for clarity. Right, histograms of firing rate modulation distributions for each phase. Triangles indicate the median values. **c**, Spontaneous firing rates across reversal learning. Pre_early_ (4.8±4.6 spikes/s, n = 148 cells), Pre_late_ (4.1±4.4 spikes/s, n = 187), Pre_expert_ (4.5±4.4, n = 97), Post_early_ (3.7±4.1, n = 306), Post_late_ (4.1±3.7, n = 342), Post_expert_ (4.3±6.1, n = 247). No significant changes were observed across learning (p > 0.05). **d**, Stimulus-evoked firing rates (positive for enhanced responses, negative for suppressed responses) to target and non-target tones during reversal learning. Target: Pre_early_ vs. Pre_late_, p = 5×10^−4^; Post_early_ vs. Post_late_, p = 5×10^−7^; Post_late_ vs. Post_expert_, p = 0.03. Nontarget: Post_early_ vs. Post_late_, p = 3×10^−5^. **e**, Distribution of tuning curves at best frequency across all learning phases (N = 10 mice). No significant changes were observed across learning (p > 0.05) or between target (solid outline) and non-target (dashed outline) tone tuning (p > 0.05); bootstrapped hypothesis test with Benjamini-Hochberg correction. Data are mean+s.e.m. *p<0.05, **p<0.01, ****p<0.0001; two-sided Mann-Whitney U test with Benjamini-Hochberg correction.

To monitor neural responses during learning we recorded from thousands of neurons in mouse ACtx as animals performed all phases of pre and post-reversal learning (n=1,327 cells, N=14 mice, **Extended Data Fig. 2a,b**). Cortical single-unit responses to task variables during learning were highly diverse and we observed a wide range of single-unit response types from ‘classically responsive’ cells that were highly modulated relative to pre-trial baseline activity to ‘non-classically responsive’ cells with relatively unmodulated firing rates throughout task performance including stimulus presentation or behavioral choice (example cells Fig. 2b; **Extended Data Fig. 3a**). To capture the continuum of heterogeneous neural response types observed during learning, we calculated a previously described ‘firing rate modulation index’^3^ comparing neural spiking responses during the stimulus or choice periods to baseline values where either positive or negative changes in spike number increase the modulation index (in units of spikes per second). Low index values (near 0 spikes/s) correspond to neurons that were unmodulated relative to baseline (‘non-classically responsive’) and larger values (≥ 3.5 spikes/s) correspond to neurons that were highly modulated (‘classically responsive’). For stimulus-related responses, neurons spanned the entire range from classically to non-classically responsive neurons during all learning phases (Fig. 2b). The range of neural responses including classically and non-classically responsive cells were recorded in superficial, middle, and deep cortical layers with a slight increase in non-classically responsive cells found in deeper layers (CRs vs. NCRs, p = 2×10^−6^; **Extended Data Fig. 2b,c;** for choice-related neural responses, **Extended Data Fig. 3b,c**).

While spontaneous activity did not change over learning (Fig. 2c; p > 0.05), evoked firing rates to target and nontarget tones were altered. During pre-reversal learning cortical responses to the target tone transiently decreased during the late phase while responses to the nontarget tone remained unchanged throughout pre-reversal learning (Fig. 2d; target early to late: p = 5×10^−4^). In contrast, cortical responses to both target and non-target tones decreased during post-reversal late and this shift was largely maintained during expert for both tones (Fig. 2d; target early to late: p = 5×10^−7^; target late to expert: p = 0.03; non-target early to late: p = 3×10^−5^). These data indicate that stimulus responses are largely suppressed during periods of rapid learning during both pre and post-reversal stages. To determine whether the tuning properties of neurons were altered during reversal learning, following a behavioral session we also measured neural responses during passive tone presentation (4–64kHz, 100ms duration, 0.5 octave steps, 70dB SPL) and did not observe systematic changes in tuning properties across learning (Fig. 2e, p > 0.05). Cortical responses in behaving animals have been shown to be modulated by movement-related signals^31^. To assess the extent to which cells were modulated by motor movements we tracked the animal’s movement during behavior while recording from ACtx using DeepLabCut^32^ (**Extended Data Fig. 4a,b**). Both classically and non-classically responsive cells were equally modulated by mouth movements, but not by nose or whisker movements indicating that movement does not uniquely modulate non-classically responsive neurons (**Extended Data Fig. 4c,d**, mouth-modulated CRs, p = 0.01; mouth-modulated NCRs, p = 0.04).

### Non-classically responsive neurons preferentially recruited during reversal learning

**Figure 3.**
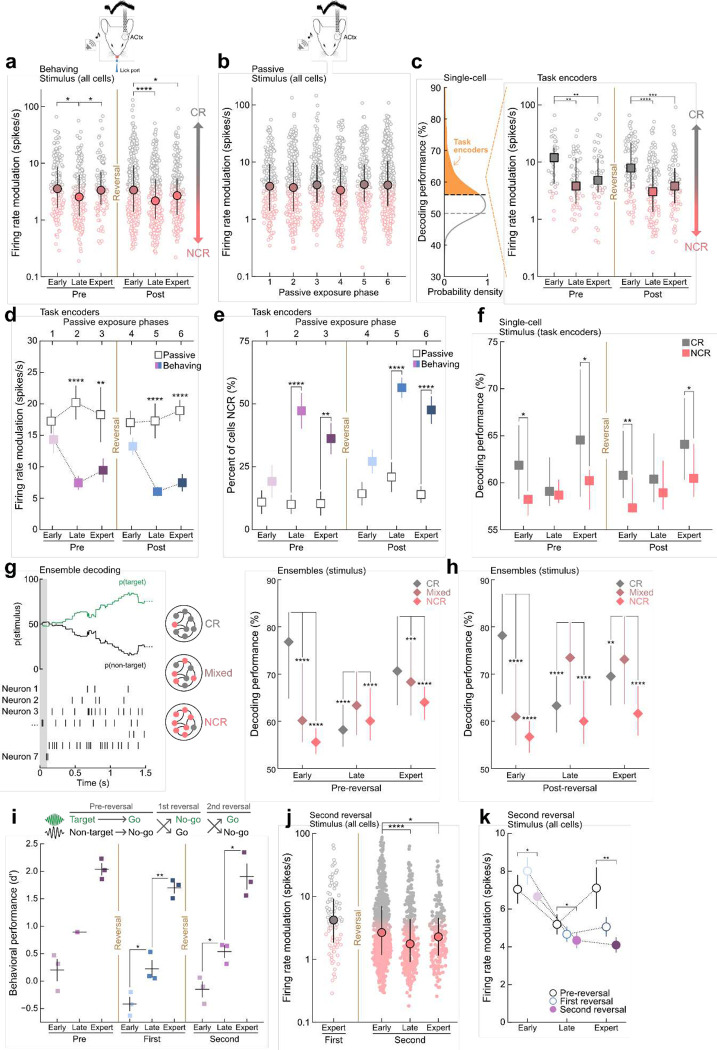
Non-classically responsive auditory cortical neurons preferentially recruited during learning. **a**, Distributions of stimulus firing rate modulations for all cells over all learning phases. Pre_early_ (range=0.2–65.7 spikes/s, n = 148 cells) vs. Pre_late_ (range=0.1–56.1, n = 187 cells), p = 0.01; Pre_late_ vs. Pre_expert_ (range=0–67.8, n = 97 cells), p = 0.04; Post_early_ (range=0.3–130.7, n = 306 cells) vs. Post_late_ (range=0.2–50.6, n = 342 cells), p = 8×10^−5^; vs. Post_expert_, (range=0.2–90.4, n = 247 cells), p = 0.01, N=14 mice. **b**, Distributions of stimulus firing rate modulations for all cells during passive exposure. Pre_early_ (range = 0.3–55.1 spikes/s, n = 198 cells), Pre_late_ (range = 0.3–132.9, n = 241 cells), Pre_expert_, (range = 0.3–144.7, n = 206 cells), Post_early_ (range = 0.3–53.3, n = 244 cells), Post_late_ (range = 0.1–107.6, n = 201 cells), Post_expert_ (range = 0.3–108.4, n = 450 cells), p > 0.05, N = 9 mice. **c**, Left, single-cell stimulus decoding performance for all cells across all learning phases, highlighting cells with significant decoding performance (‘task encoders’), n = 448/1,327 cells; test against one-sided 95% confidence interval set by below-chance decoding neurons. Right, distributions of stimulus firing rate modulations for task-encoding cells across learning. Pre_early_ (n = 37 cells) vs. Pre_late_ (n = 51 cells), p = 0.002; vs. Pre_expert_ (n = 50 cells), p = 0.01; Post_early_ (n = 93 cells) vs. Post_late_ (n = 137 cells), p = 1×10^−7^; vs. Post_expert_ (n = 80 cells), p = 2×10^−4^. **d**, Mean stimulus firing rate modulation for task-encoding cells across learning for cells from **c** vs. stimulus-encoding cells in passive control animals (n = 364 cells); Pre_late_, p = 2×10^−6^; Pre_expert_, p = 0.003; Post_late_, p = 2×10^−7^; and Post_expert_, p = 7×10^−10^. Data are mean+s.e.m. **e**, Percent of cells from **d** that are stimulus task-encoding non-classically responsive cells (NCR) across learning vs. stimulus-encoding passive control animals (Pre_late_, p < 4×10^−6^; Pre_expert_, p = 0.001; Post_late_, p < 4×10^−6^; Post_expert_, p < 4×10^−6^), bootstrapped hypothesis test with Benjamini-Hochberg corrections. Data are mean+s.e.m. **f**, Single-cell stimulus decoding performance of task-encoding neurons. CR, classically responsive cells; NCR, non-classically responsive cells from **c** (Pre_early_, p = 0.01; Pre_late_, p = 0.8; Pre_expert_, p = 0.01; Post_early_, p = 0.001; Post_late_, p = 0.1; Post_expert_, p = 0.02). **g**, Left, example ensemble stimulus decoding performance for an ensemble on a single trial. Shaded region indicates tone duration. CR ensembles, classically responsive ensembles; Mixed, ensembles comprised of both CRs and NCRs; NCR ensembles, non-classically responsive ensembles. Right, ensemble stimulus decoding performance for all cells during pre-reversal. Pre_early_ (CR vs. mixed, p = 3×10^−54^; vs. NCR, p = 1×10^−72^, n = 1,307 ensembles; Pre_late_ (mixed vs. CR, p = 1×10^−10^; vs. NCR, p = 6×10^−6^ n = 1,145 ensembles); Pre_expert_ (CR vs. mixed, p = 0.0001; vs. NCR, p = 2×10^−13^, n = 836 ensembles). **h**, Ensemble stimulus decoding performance for all cells during post-reversal. Post_early_ (CR vs. mixed, p = 6×10^−92^; vs. NCR, p = 1×10^−109^, n = 2,258 ensembles); Post_late_ (mixed vs. CR, p = 7×10^−25^; vs. NCR, p = 3×10^−94^; n = 2,486 ensembles); Post_expert_ (mixed vs. CR, p = 0.006; vs. NCR, p = 4×10^−35^, n = 1,122 ensembles). **i**, Behavioral performance for animals that underwent a second reversal. Pre_early_ (d’ = 0.2±0.2, N = 3 mice); Pre_late_ (d’ = 0.9, N = 1 mouse); Pre_expert_ (d’ = 2.0±0.1, N = 3 mice); First_early_ (d’ = −0.4±0.1, N = 3 mice) vs. First_late_ (d’ = 0.2±0.2, N = 3 mice), p = 0.03; First_late_ vs. First_expert_ (d’ = 1.6±0.1, N = 3 mice), p = 0.005; Second_early_ (d’= −0.1±0.2, N = 3 mice) vs. Second_late_ (d’= 0.5±0.1, N = 3 mice), p = 0.02; Second_late_ vs Second_expert_ (d’= 1.9±0.3, N = 3 mice), p = 0.01; two-sided independent t-test with Benjamini-Hochberg corrections. Data are mean+s.e.m. **j**, Distribution of stimulus firing rate modulations across learning for the expert phase of the first reversal (Post_expert_) and all phases during the second reversal. Second_early_ (n = 672 cells) vs. Second_late_ (n = 329 cells, p = 3×10^−5^); vs. Second_expert_ (n = 194, p = 0.03). **k**, Mean stimulus firing rate modulations during pre-reversal, first-reversal, and second reversal. Pre_early_ vs. Second_early_, p = 0.04; Pre_late_ vs. Second_late_, p = 0.03; Pre_expert_ vs. Second_expert_, p = 0.004, s.e.m. shown. Data are median+i.q.r except where otherwise mentioned. *p<0.05, **p<0.01, ***p<0.001, ****p<0.0001 two-sided Mann-Whitney U test with Benjamini-Hochberg correction.

We wondered how these diverse neural populations emerge and evolve during learning. To examine this, we compared the distribution of response profiles during early learning, late learning, and expert phases. Although single-unit responses were highly heterogeneous within a given learning phase we observed distinct population-level changes in neural firing properties across learning. We found that the stimulus firing rate modulations significantly decreased during late learning in both phases when compared to early learning stages in each phase, respectively (Fig 3a, Pre_early_ vs. Pre_late_: p = 0.01; Post_early_ vs. Post_late_: p = 8×10^−5^). Using a statistically defined threshold to identify classically and non-classically responsive cells (see Methods section “Discrete characterization of classically and non-classically responsive units”; **Extended Data Fig. 5a**), we observed a 24.4% increase in the number of non-classically responsive cells during late learning in the pre-reversal phase and a 25.5% increase during post-reversal (**Extended Data Fig. 5b;** for choice-related activity, **Extended Data Fig.7a-d).** These dynamics resulted in a population-level shift towards non-classically responsive activity during late learning, where the greatest gains in behavioral performance were observed (i.e. the rapid learning phase, Fig.1d). To confirm that these changes were a result of learning, we passively exposed a separate cohort of control animals to the same stimuli matched for exposure duration as trained animals. Passively-exposed controls did not demonstrate systematic changes in their firing rate modulation across exposure days indicating that the population-level dynamics observed in trained animals are learning-related (Fig 3b, N=9 animals, p > 0.05).

Next, we examined the extent to which task information (stimulus or behavioral choice) is encoded in single-neurons and how it evolves over learning. Applying a previously described single-trial, spike-timing-dependent Bayesian decoder^3,4^ on single-neurons, we observed that stimulus decoding performance varied widely (~45–90%) with the majority of cells decoding better than chance (888/1,327 cells, **Extended Data Fig. 5c**). Stimulus decoding performance also significantly improved over the course of learning in both pre-and-post-reversal learning phases (**Extended Data Fig. 5c**, Pre_early_ vs. Pre_expert_, p = 2×10^−6^; Post_early_ to Post_expert_, p = 3×10^−5^). To assess the statistical significance of these results, we tested decoding performance on a control data set using synthetically-generated trials that preserved trial length but randomly sampled ISIs with replacement from those observed during a behavioral session. In the synthetic controls, decoding performance remained at chance and did not improve over learning (**Extended Data Fig**. **5d**, p > 0.05)

We then determined the fraction of cells that had stimulus decoding performance signficantly greater than chance (Fig. 3c, left, >56% decoding performance; threshold set using one-sided 95% confidence interval around chance, n = 448/1,327 cells) and next examined the dynamics of this highly-informative task-encoding subpopulation. As in the overall population, we observed a wide range of firing rate modulations within learning phases, and a significant decrease in firing rate modulation during late learning in both phases (Fig. 3c, right, Pre_early_ vs. Pre_late_, p = 0.002; Post_early_ vs. Post_late_, p = 1×10^−7^) resulting in a larger shift towards non-classically responsive activity compared to the overall population (Fig. 3a). We also applied our decoder to the passive control animals and identified the fraction of cells that had stimulus decoding performance significantly higher than chance. Comparing the firing rate modulation of the task-encoding cells in trained animals to the passive control animals we found that the firing rate modulation was significanty lower throughout learning in trained animals, (Fig. 3d, Pre_late_, p = 2×10^−6^; Pre_expert_, p = 0.003; Post_late_, p = 2×10^−7^; and Post_expert_, p = 7×10^−10^) with a larger shift than what was observed in the general population (**Extended Data Fig. 5e**; for choice-related decoding, **Extended Data Fig.7e-j**).

We wanted to next further examine these population-level dynamics observed during learning. We found that the proportion of task-encoding non-classically responsive neurons significantly increased during late learning in both phases (Pre: 147% increase; Post: 107% increase, **Extended Data Fig. 5f**) coinciding with the greatest improvements in behavioral performance (Fig.1d) and was maintained during expert learning in both phases relative to passive controls (Fig. 3e, Pre_late_, p < 4×10^−6^; Post_late_, p < 4×10^−6^). This shift towards non-classically responsive activity in the task-encoding population was much larger than what was observed in the general population (**Extended Data Fig. 5b,e**). These data demonstrate that non-classically responsive neurons are preferentially recruited during learning, particularly when learning is most dynamic.

Notably, we did not observe a monotonic increase in decoding performance over learning for the task-encoding cells. Instead we found that classically-responsive cells had significantly higher decoding performance during early and expert learning phases, but not during late learning when the greatest gains in behavioral performance occur (Fig. 3f, Pre_early_, p = 0.01; Pre_late_, p = 0.8; Pre_expert_, p = 0.01; Post_early_, p = 0.001; Post_late_, p = 0.1; Post_expert_, p = 0.02). These dynamics resulted in matched decoding performance for task-encoding classically and non-classically responsive cells during late learning at the single-cell level and suggested that a population-level analysis might shed light on how task-information was being encoded in auditory cortical circuits.

To examine how task information is represented at the population level, we decoded from ensembles of simultaneously recorded cells that included varying fractions of classically and non-classically responsve cells (Fig. 3g, left, CR ensembles = 70–100% classically responsive cells; Mixed ensembles = 50–70% non-classically responsive cells; NCR ensembles = 80–100% non-classically responsive cells). We found that during pre-reversal late, mixed ensembles comprised of both classically and non-classically responsive cells had the highest stimulus decoding performance (Fig. 3g, right, Mixed vs. CRs, p = 1×10^−10^; Mixed vs. NCRs, p = 6×10^−6^) while during pre-reversal early and expert, ensembles comprised of mostly classically-responsive neurons had the highest decoding performance (Fig. 3g, **right**; Pre_early_, CRs vs. Mixed, p = 3×10^−54^; CRs vs. NCRs, p = 1×10^−72^; Pre_expert_, CRs vs. Mixed, p = 0.0001; CR vs. NCR, p = 2×10^−13^; **Extended Data Fig**. **5g**). Examing post-reversal learning, population-level decoding revealed that beginning at late learning and continuing to expert, mixed ensembles encoded significantly more task information than homogenous ensembles of either type (Fig. 3h, Post_late_ CRs vs. Mixed: p = 7×10^−25^; NCRs vs. Mixed: p = 3×10^−94^; Post_expert_ CRs vs. Mixed: p = 0.006; NCRs vs. Mixed: p = 4×10^−35^; **Extended Data Fig**. **5h**). Classically-repsonsive ensembles had the highest decoding performance early in learning, similar to pre-reversal early (Fig. 3h, CRs vs. Mixed, p = 6×10^−92^; CRs vs. NCRs, p = 1×10^−109^). These data indicate that as animals undergo reversal learning, mixed ensembles comprised of mostly non-classically responsive cells are recruited at a population-level to encode task-relevant information and emerge as a functional unit critical for learning.

We next wondered how generalizable this preferential recruitment of non-classically responsive cells is and recorded from the ACtx while animals were reversed a second time (Fig. 3i). During the second reversal we observed a significant decrease in the firing rate modulation and an increase in the percent of cells that are non-classically responsive during late learning consistent with the first-reversal results (Fig. 3j,k Second_early_ vs. Second_late_, p = 3×10^−5^; vs. Second_expert_, p = 0.03; **Extended Data Fig. 5i,**). There was also a significant increase in the percent of non-classically resposive cells during the second reversal compared to the first indicating that as the repertoire of reversals increase so does the population of non-classically responsive cells which may be related to the animal’s ability to generalize across reversals (**Extended Data Fig. 5i,** Second_expert_ vs. Pre_expert_, p < 4×10^−6^; vs. First_expert_, p = 0.01). These results suggest that the initial shift towards non-classically responsive activity is specific to late learning and is present during initial task acquisition, and multiple reversals. Notably, on the first day of reversal we recorded from the same cells before and after the rule switch and did not observe a significant change in either the spontaneous firing rate, or the evoked firing rates to both tones immediately following reversal (**Extended Data Fig. 6a-c,** p > 0.05).

### M2 inputs recruit non-classically responsive cells in auditory cortex to enable behavioral flexibility

**Figure 4.**
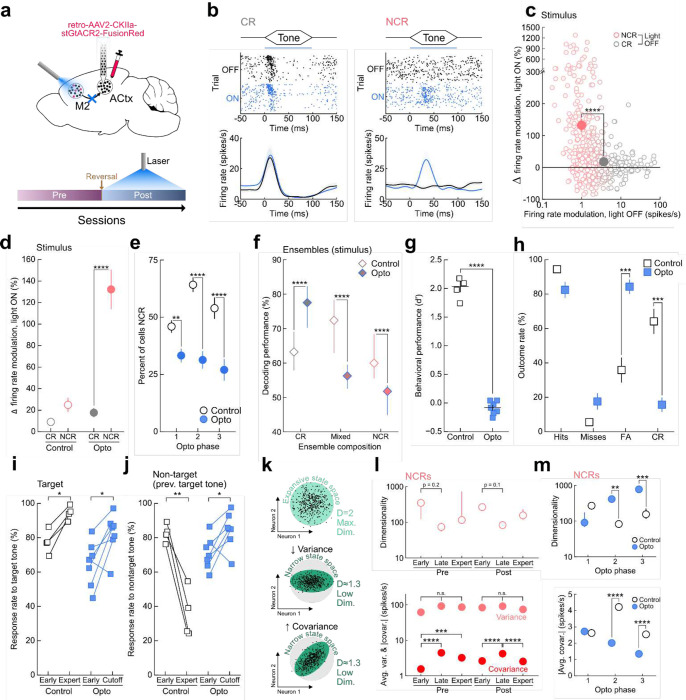
M2 recruits non-classically responsive cells in auditory cortex to enable reversal learning. **a**, Top, bilateral photoinhibiton of M2 inputs to ACtx during post-reversal learning. Bottom, photoinhibition schedule during post-reversal. **b**, Example raster and PSTHs during photoinhibition for a classically-responsive cell (CR) and a non-classically responsive cell (NCR), blue bar = light ON. Horizontal bar in raster, laser duration. Lines in PSTH, mean firing rate; shading, s.e.m. **c**, Change in stimulus firing rate modulation for all cells during photoinhibition. CR (n = 295 cells) vs. NCR (n = 227 cells), p = 3×10^−14^; two-sided Mann-Whitney U test with Benjamini-Hochberg correction. **d**, Stimulus firing rate modulation for all classically responsive (CR, gray) and non-classically responsive cells (NCR, red). Animals expressing inhibitory opsin that underwent laser stimulation, referred to as Opto. Opto CRs (n = 295 cells) vs. opto NCRs (n = 227 cells), p = 3×10^−14^; control CRs (n = 135 cells) vs. control NCRs (n = 153 cells), p = 0.2; two-sided Mann-Whitney U test. **e**, Percent of all cells that are stimulus non-classically responsive (NCR) during optogenetic manipulation. The duration of photoinhibition was divided into 3 phases and compared to the early, late, and expert learning phases observed in control animals (Opto, n = 522 cells; control, n = 675 cells), Phase 1, p = 1×10^−3^; Phase 2, p < 4×10^−6^; Phase 3, p = 6×10^−5^; bootstrapped hypothesis test with Benjamini-Hochberg corrections. **f**, Ensemble stimulus decoding performance during Opto phase 2 (the equivalent of Post_late_). Classically-responsive ensembles (Opto, n = 966 ensembles vs. control, n = 205 ensembles, p = 3×10^−40^); Mixed ensembles comprised of both CRs and NCRs (Opto, n = 308 ensembles vs. control, n = 644 ensembles, p = 9×10^−69^); Non-classically responsive ensembles (Opto, n = 22 ensembles vs. control, 917 ensembles, p = 1×10^−9^). Data are median+i.q.r; two-sided Mann-Whitney U test with Benjamini-Hochberg correction. **g**, Behavioral performance on the last three sessions of behavior for opto (d’ = −0.08±0.1, N = 7 mice) and controls (d’ = 2.0±0.1, N = 4 mice), p = 2×10^−9^. **h**, Trial outcome rates on the last three sessions of behavior of animals from **g**. Opto vs. controls for false alarms and correct rejects, p = 2×10^−4^; for hits and misses, p = 0.08. **i**, Response rates on target trials at start of post-reversal (‘start’, first 3 sessions) vs. the last three sessions (‘expert’ for control, ‘end’ for opto); opto, p = 0.02; control, p = 0.01. **j**, Response rates on non-target trials at start of post-reversal vs. the last three sessions of behavior; opto, p = 0.03; control, p = 0.006. **k**, Top, high single-unit variance and low pairwise covariance produces activity with the highest possible dimensionality, covering the largest number of possible states. Middle, decreasing single-unit variance leads to a decrease in dimensionality and a correponsding narrowing of the possible state space. Bottom, increasing pairwise covariance leads to a decrease in dimensionality and a corresponding narrowing of the possible state space without decreasing single-unit variance. **l**, Top, dimensionality of activity of all non-classically responsive neurons over learning. Dimensionality decreases during the transition from early to late learning. Pre_early_ vs. Pre_late_, p = 0.2; Post_early_ vs. Post_late_, p = 0.1; bootstrapped hypothesis test with Benjamini-Hochberg corrections. Bottom, average variance and absolute covariance between non-classically responsive neurons over learning. Light red, variance, p > 0.05. Dark red, absolute covariance, Pre_early_ vs. Pre_late_, p = 3×10^−31^; Pre_early_ vs. Pre_expert_, p = 1×10^−9^; Post_early_ vs. Post_late_, p = 3×10^−23^; Post_late_ vs. Post_expert_, p = 1×10^−9^; two-sided student’s t-test with Benjamini-Hochberg corrections. **m**, Top, dimensionality of activity of non-classically responsive neurons in photoinhibited opto vs. control animals during post-reversal. Phase 2, p = 0.003; Phase 3, p = 0.0004; bootstrapped hypothesis test with Benjamini-Hochberg corrections. Bottom, average absolute covariance between non-classically responsive neurons in photoinhibited opto vs. control animals during post-reversal. Phase 2, p = 1×10^−5^; Phase 3, p = 9×10^−7^; two-sided student’s t-test with Benjamini-Hochberg corrections. Data are mean+s.e.m except where otherwise mentioned. *p<0.05, **p<0.01, ***p<0.001, ****p<0.0001, two-sided independent t-test with Benjamini-Hochberg correction.

We hypothesized that reversal learning could be under top-down control from secondary motor cortex (M2) and optogenetically inactivated M2→ACtx projection neurons during post-reversal training (Fig. 4a; **Extended Data Fig. 8a,b**) while recording neural responses in ACtx. M2 has previously been shown to modulate responses in ACtx during locomotion via direct feedback projections^33–35^. Surprisingly, photoinibition of M2→ACtx projection neurons during tone presentation selectively modulated non-classically responsive neurons in ACtx when compared to both laser OFF trials (Fig. 4b,c; **Extended Data Fig. 8c,d**) and sham control animals (NCRs: 132% change in firing rate modulation, Fig. 4d; **Extended Data Fig. 9a**). By contrast, photoinhibition only moderately altered the activity of classically-responsive cells (Fig. 4b–d; CRs: 18% change in firing rate modulation). Examining the stimulus-selectivity of non-classically responsive cells that were transformed into classically-responsive cells following photoinhibition revealed that M2 inputs suppress target and non-target tuned neurons similarly, indicating that the selective modulation of non-classically responsive cells in ACtx is stimulus-independent (**Extended Data Fig. 9b**). This demonstrates that M2 inputs do not simply suppress the previous target tone (i.e., the current nontarget tone) but broadly suppresses stimulus-evoked activity in ACtx. The duration of photoinhibition was divided into 3 phases to examine how the course of photoinhibition altered neural responses in ACtx (opto phase 1 = first 2 days of photoinhibition; opto phase 2 = days 3–4; opto phase 3 = 5–7 days). Examining all phases of photoinhibition, we found that silencing M2 inputs prevented the recruitment of non-classically responsive cells during post-reversal (Fig. 4e, Phase 1, p = 1×10^−3^; Phase 2, p < 4×10^−6^; Phase 3, p = 6×10^−5^). In order to examine how silencing M2 inputs altered the computation of local ACtx circuits we decoded stimulus information from ensembles of ACtx neurons during photoinhibition. We found that photoinhibition impaired stimulus decoding performance and prevented the formation of highly-informative mixed and non-classically responsive ensembles during opto phase 2, the equivalent of late learning (Fig. 4f, CRs, p = 3×10^−40^; NCRs, p = 1×10^−9^; Mixed, p = 9×10^−69^; **Extended Data Fig. 9c)**. These results indicate that M2→ACtx projections preferentially recruit non-classically responsive neurons into diverse task-encoding ensembles, and photoinhibition of this pathway preferentially modulates non-classically reponsive cells in ACtx.

We next examined the effect that silencing M2→ACtx projection neurons had on reversal learning. Photoinhibition of M2 inputs during tone presentation resulted in impaired post-reversal learning compared to controls (Fig. 4g, Controls: d’ = 2.0±0.1; Opto: d’ = −0.08±0.1; **Extended Data Fig. 8e**). Interestingly, photoinhibition during post-reversal learning resulted in signficantly higher false alarm rates and lower correct reject rates without affecting the corresponding hit and miss rates (Fig. 4h, N = 7 opto animals, N = 4 control animals; false alarms p = 2×10^−4^, hits p = 0.08). Notably, the behavioral impairments were specific to the non-target tone as photoinhibited animals were able to learn to correctly respond to the new target tone (Fig. 4i, p = 0.02). Thus, while photoinhibited animals are able to learn to respond correctly to the new target tone, they do not learn to suppress their responses to the previous target tone (Fig. 4j, p = 0.03). This indicates that M2→ACtx projections may be involved in the ability to override previously learned associations. We wondered whether these behavioral changes were simply a result of photoinhibition inducing a motor bias (always respond with a go-response) and examined how the response rate to target and nontarget tones changes from the start of photoinhibition to the end. We found that at the start of photoinhibiton, go responses were not significantly higher in opto animals when compared to control animals indicating that inhibiting M2 inputs does not simply result in a motor response (**Extended Data Fig. 9d**). In addition, both the hit and false alarm rates increase during the course of photoinhibition (Fig. 4i,j, opto, hits: p = 0.02; false alarm: p = 0.03) suggesting that the behavioral impairment is not related to an induced motor bias as we would expect this to be expressed from the first day of photoinhibition.

Finally, we wondered how non-classically responsive cells in ACtx affect circuit dynamics to generate flexible behavior and investigated the mechanism by which these cells enable reversal learning. To examine this, we calculated the dimensionality of population activity^36^ to assess how individual neurons influence global network dynamics during learning. Dimensionality is a single metric which describes the number of independent modes of activity observed in the entire population (Fig. 4k). It has been shown to capture important computational properties of the network^37,38^ and is specifically relevant for understanding the dynamics responsible for steady-state behaviors^21^. In asymptotically large networks, the network dimensionality depends only on the variance and covariance of network units, equation (12). The single-unit variance serves to expand the scope of possible network states (increases dimensionality; Fig. 4k, middle) while pairwise covariance reduces the global states available to the network (decreases dimensionality; Fig. 4k, bottom). We observed a decrease in dimensionality of non-classically responsive cells from early to late learning (when behavioral changes were the most rapid) during both pre- and post-reversal (Fig. 4l, top). These changes were driven by an increase in the pairwise correlations between non-classically responsive cells during late learning rather than a change in the variance of individual units (Fig. 4l, bottom). By contrast, optogenetically inactivating M2 inputs decorrelated non-classically responsive activity in ACtx and correspondingly increased network dimensionality, demonstrating that M2 constrains the overall dynamics in ACtx and restricts activity to a narrow subspace during reversal learning (Fig. 4m). As previously described, inactivation of M2 also impairs reversal learning by increasing the false alarm rate to the previous target tone (Fig. 4g,h). This suggests that top-down inputs onto non-classically responsive cells in ACtx collectively constrain network states to a new space to allow for the formation of new stimulus-reward associations while preventing the network from reverting back to now irrelevant subspaces and perseverating on previously learned stimulus-reward contingencies (**Extended Data Fig. 9e**).

Notably, while changes in the dimensionality of classically responsive cells were also driven by differences in pairwise covariance (**Extended Data Fig. 9f**), they were less pronounced in general – consistent with observation that M2 preferentially modulates the activity of non-classically responsive units (Fig. 4c). Unlike non-classically responsive cells, the largest changes in dimensionality occur in the transition to the expert phase (both pre- and post-reversal) after the largest improvements in performance have concluded (**Extended Data Fig. 9f**). Photoinhibition increased correlations between classically responsive cells during opto phase 3 demonstrating that while M2 inputs correlate non-classically responsive cells during learning they de-correlate the activity of classically responsive neurons (**Extended Data Fig. 9g**). Although non-classically responsive cells restrict the subspace of dynamics during rapid learning, the dynamics of classically responsive cells may be important for efficiently driving behavior during expert performance after the stimulus-reward contingencies have been remapped.

## Discussion

In summary, flexible sensory-guided behaviors result from the preferential recruitment of non-classically responsive neurons in sensory cortex by top-down inputs in M2. It is unclear how secondary motor inputs specifically modulate non-classically responsive neurons in ACtx. We speculate that parvalbumin inhibitory neurons in ACtx could allow for this specificity. As previously shown, stimulus-evoked responses in excitatory auditory cortical neurons are suppressed by inputs from M2^33,34^. These inputs from M2 synapse onto local parvalbumin positive neurons in ACtx mediating the suppression of stimulus-evoked responses during locomotion. During flexible behaviors, M2 may synapse onto parvalbumin positive interneurons in ACtx which selectively innervate non-classically responsive neurons allowing for feedforward inhibition in this specific subpopulation. This parallel circuit allows some cells to remain tuned (i.e. the classically responsive cells) which may be important for stable representations of the stimulus while allowing a second subpopulation to dynamically adapt to changing task contingencies (i.e the non-classically responsive cells) to enable learning. This heterogeneity in neural responses may be important for a variety of adaptive behaviors including sequence learning^17^, multisensory decision-making^5^, contextual modulation^13,15^, task-switching^7^, and rule-encoding^4^ in both artificial and real biological systems.

## Methods

All animal procedures were performed in accordance with National Institutes of Health standards and were conducted under a protocol approved by the University of Pittsburgh School of Medicine Institutional Animal Care and Use Committee.

### Surgical preparation

Adult 2–3 month old C57Bl/6 female and male mice were anesthetized with isoflurane (3% during induction, 2% during surgery), and a custom-designed stainless steel headpost was affixed to the skull with dental cement (Metabond). Each animal was allowed to recover for 7+ days.

### Auditory reversal learning behavioral paradigm

Mice were trained on an auditory reversal learning task following 4 days of water-restriction. Behavioral events (stimulus delivery, water delivery, and lick detection) were monitored and controlled by custom-written programs in MATLAB that interfaced with an RZ6 processor (Tucker-Davis Technologies). Auditory stimuli were played through an electrostatic speaker (Tucker-Davis Technologies). Animals were first habituated to head-fixation and then trained to associate licking with a water reward. Animals were then trained on the ‘pre-reversal’ part of the task to respond to the target tone (11.2 kHz) by licking for water reward (‘hit’) and to withhold responses to the non-target tone (5.6 kHz; ‘correct reject’). Once animals reached behavioral criteria (percent correct: ≥70%, and d’: ≥1.5) they were trained on the ‘post-reversal’ part of the task where a rule switch was implemented by reversing which tone is rewarded (new target tone =5.6 kHz; new nontarget tone =11.2 kHz). Auditory stimuli were 100 ms duration, 3 ms cosine on/off ramps, at 70dB SPL. Animals had 2.5 seconds to respond, only hits were rewarded, and only incorrect responses to the nontarget tone (‘false alarm’) resulted in a 7 sec timeout.

To identify learning phases, we calculated the range of behavioral performance during pre- and post-reversal. ‘Early’ learning was defined as less than 40% progress from minimum to maximum d’ performance, ‘late’ learning was defined as greater than 40% progress towards maximum d’ performance, and ‘expert’ was defined as performance with d’: ≥1.5 and percent correct: ≥70%. Learning curve slopes for each phase were calculated by taking the slope of a linear least squares regression of all sessions in the phase and, in the case of non-expert phases, the first session of the next phase.

### Chemogenetic inactivation experiments

For chemogenetic inactivation experiments using inhibitory DREADDs, a small craniotomy was made over auditory cortex (1.75 mm anterior to the junction of the temporal ridge and the lambdoid suture) and pAAV-CaMKIIa-hM4D(Gi)-mCherry (Addgene) was bilaterally injected (0.56μL per hemisphere; 50nL/min injection speed) using a 5μL Hamilton syringe (33-gauge needle). Mice were given 2–3 weeks for viral expression and recovery after which behavioral training began. To activate the inhibitory hM4D(Gi) receptor, clozapine N-oxide (CNO, i.p. 5mg/kg, Tocris) was injected 30 min prior to the start of the behavioral session. For inactivation during pre-reversal training, mice were injected with CNO from day 1 of pre-reversal until day 10 or once animals reached expert behavioral criteria. The duration of inactivation was determined by taking one standard deviation above the average number of days control animals take to learn the pre-reversal stage (average plus standard deviation: 6.80±3.0 days). For inactivation during post-reversal training, mice were first trained on pre-reversal and were injected with CNO on the first day of post-reversal and the following 12 days during post-reversal training corresponding to one standard deviation above the average number of days control animals take to learn the post-reversal stage (average plus standard deviation: 9.75± 2.60 days).

### *In vivo* electrophysiology during learning and passive exposure

For *in vivo* electrophysiological experiments, a craniotomy was made over auditory cortex (located 1.75 mm anterior to the lambdoid suture). A stainless steel ground wire was placed under the skull over the pial surface, and a stainless steel reference wire was placed adjacent to the craniotomy site. All recordings were made in a single-walled sound isolation booth (Eckel). Following recovery, animals were head-fixed and a 64-channel silicon probe (Cambridge Neurotech, H5 probes with a single shank or H6 probes with dual shanks) was inserted into auditory cortex using a micromanipulator (Sutter MP-285). After the probe was removed, the craniotomy site was covered with a silicone gel (Dura-Gel, Cambridge NeuroTech) and silicone sealant (Kwik cast). Recordings were made daily for 7–10 days and were staggered across learning phases. A random recording site was chosen each day to avoid oversampling of a given tonotopic area. Neural signals were amplified, sampled at 30 kS/s bandpass filtered between 250 Hz and 5 kHz, digitized, and stored for offline analysis (CerePlex Direct, Blackrock Microsystems). Single-units were identified offline using Kilosort 2.5 spike sorting software (Pachitariu et al 2016, https://github.com/MouseLand/Kilosort). First, electrophysiological data were automatically spike sorted using Kilosort and then manually curated using ‘phy’ (Rossant et al., 2016; https://github.com/cortex-lab/phy). Each set of spikes were manually inspected, and were merged depending on spike waveform similarity, cross-correlogram features, and drift patterns in spiking activity. Sets of spikes that contained multiple cleanly separable units were clustered based on template features. Units with refractory period violations (< 1ms) and or significant non-stationarity were excluded from analysis. To be included for analysis, cells had to have an average firing rate of 0.5 spikes/sec or greater.

For passive exposure recordings, we passively exposed naïve animals to the same stimuli as behaving animals (11.2 kHz and 5.6 kHz pure tones randomly presented with 3–7 sec inter-tone intervals, 100 ms duration, 3 ms cosine on/off ramps, at 70dB SPL, 400 repetitions) for up to 21 days. Recordings were staggered, starting from day 1 of exposure to day 7, days 8–14, and days 15–21 to match recording timepoints in the behaving animals. This allowed us to have a point-to-point correspondence of passive exposure with active exposure recordings, with passive exposure phase 1 (recording days 1–4) corresponding to pre-reversal early, passive exposure phase 2 (recording days 5–6) corresponding to pre-reversal late, passive exposure phase 3 (recording days 7–8) corresponding to pre-reversal expert, passive exposure phase 4 (recording days 9–12) corresponding to post-reversal early, passive exposure phase 5 (recording days 13–15) corresponding to post-reversal late, and passive exposure phase 6 (recording days 16–21) corresponding to post-reversal expert. The number of recording days included in each passive exposure phase corresponds to the average number of days trained animals spent in a given learning phase.

For tuning curve recordings, after a behavioral session we passively exposed mice to a range of pure tone stimuli (4–64 kHz randomly presented every 1.4 s, 1 octave spacing, 3 ms cosine on/off ramps, 70 dB SPL, 100 ms duration, 450 total repetitions). Single-unit recordings from auditory cortex were made during passive presentation using 64-channel silicon probes in animals undergoing both pre-and-post reversal training.

### Spontaneous and evoked firing rate calculation

The spontaneous average firing rate was calculated by averaging spikes in a 150ms time window immediately prior to tone onset on each trial.

The stimulus evoked firing rate, R, for each cell was calculated by comparing the spontaneous firing rate, $R_{bl}$, to the firing rate in a 200 ms window including tone presentation (100 ms) and extending 100 ms beyond tone offset, $R_{st\;i}$, to capture both tone onset and offset responses. A 50-ms sliding window within the 200ms time window following tone presentation, position represented by i, was used to capture the largest difference in the firing rate, relative to the baseline spontaneous rate. Both enhanced and suppressed responses were captured with this metric.


(1) $$j=\underset{i}{argmax}\left|R_{st_{i}}-R_{bl}\right|\;R=R_{st_{j}}-R_{bl}$$

In order to quantify the tuning properties of individual cells to the target and non-target tones, a tone selectivity index, SItone, for each cell was calculated. We calculated the absolute value of the evoked firing rate on target trials, $R_{T}$, and on non-target trials, $R_{NT}$. We calculated the ratio of the difference between $R_{T}$ and $R_{NT}$ to their sum,

(2) $${SI}_{\text{tone}}=\frac{R_{T}\;-\;R_{NT}}{R_{T}\;+\;R_{NT}}$$

A value of 0 indicates equal modulation by both tones, values between 0 and 1 indicate tuning to target tone, and values between 0 and −1 indicate tuning to non-target tone.

### Characterization of response profiles using a continuous measure (firing rate modulation)

To comprehensively characterize spiking responses, we used a continuous measure of responsiveness which generalizes the binary classification (classically vs. non-classically responsive) we used previously^4^. Our continuous measure of responsiveness quantified the degree to which a cell exhibited firing rate changes during both the stimulus and choice periods. For the stimulus firing rate modulation, we calculated the trial-averaged change in firing rate for each neuron during stimulus presentation, $R_{st}$, relative to a baseline period preceding stimulus onset, $R_{bl}$. To calculate $R_{st\;i}$, we used a 50 ms sliding window from stimulus onset up to 200 ms post-stimulus, position represented by i, to capture offset responses. We found the 50-ms bin with maximum difference from baseline and used that as the stimulus period since onset and offset responses to tone stimuli were brief (on the order of 50 ms) and could have varying latencies. To calculate $R_{bl}$, we used a 150-ms window immediately preceding tone presentation. The firing rate modulation, R, is the maximum absolute value of the difference in firing rate between the stimulus and baseline period,

(3) $$R=\underset{i}{max}\;\left|R_{st_{i}}-R_{bl}\right|$$

For the choice firing rate modulation, the trial-averaged change in firing rate during the behavioral choice, $R_{\text{ch}}$, was calculated using a 100-ms window centered on the behavioral response on ‘go’ trials and a 100-ms window centered on the average behavioral response on ‘no-go’ trials. The firing rate modulation, R, is the absolute value of the difference in firing rate between the choice and baseline period,

(4) $$R=\left|R_{ch}-R_{bl}\right|$$

This measure captures the detailed evoked or suppressed firing rate modulation of individual units during both the stimulus and response periods. It provides information about the degree to which a unit is classically responsive such that values close to 0 are only possible when a unit is non-classically responsive (e.g., a unit with firing rate modulation of 0.1 spikes/s would be classified as a non-classically responsive unit whereas we would consider 5 spikes/s as highly classically responsive).

### Discrete characterization of classically and non-classically responsive units

Statistical identification of classically and non-classically responsive units followed previously described methods^4^. We used two positive statistical tests for classical and non-classical responses to establish the presence or lack of responses during the stimulus and response periods. The tests compared the number of spikes during each of these windows to inter-trial baseline. Given that spike counts are discrete, bounded, and non-normal, we used subsampled bootstrapping to evaluate the mean change in spikes during tone presentation or the response period. We subsampled 90% of the spike count changes from baseline, calculated the mean of these values, and repeated this process 5,000 times to construct a distribution of means. If 95% of the subsampled mean values were outside of −0.1 and 0.1, we considered the cell classically responsive (p<0.05). If 95% of the subsampled mean values were between −0.1 and 0.1, we considered the cell non-classically responsive (p<0.05). We fitted a separating threshold to the distributions of firing rate modulations for the statistically defined classically and non-classically responsive cells for both the stimulus and choice periods using a support vector machine (**Extended Data Figs. 5a, 7d**). We used these thresholds to classify all cells as either classically or non-classically responsive based on their firing rate modulations. Neurons were classified as classically or non-classically responsive for either stimulus or choice depending on the variable of relevance for each analysis.

### Single-trial, single-cell, ISI-based Bayesian decoding

We applied a single-trial Bayesian ISI-based trial-by-trial decoding algorithm previously described^4^. The posterior probability of a given stimulus (or choice) was estimated using Bayes’ rule from the observed likelihood of a sequence of ISIs given the stimulus (or choice) under the simplifying assumption that the probability of each observed ISI in a spike train is independent given the task condition. The probability density function for observing an ISI on target/nontarget trials (or go/no-go trials) was inferred (p(target), p(non-target), p(go), and p(no-go)) via kernel density estimation with the bandwidth set by cross-validated maximum likelihood estimation. These probability density functions were used to infer the probability of a stimulus and choice from the observed ISIs, {ISI}, on a new trial taken via Bayes rule (assuming statistical independence between the ISIs observed) with the probabilities of stimulus (or choice) were assumed to be flat (e.g. 50% target, 50% non-target). This process was conducted using 10-fold cross validation repeated for 500 iterations.


(5) $$p(\text{stimulus}|\{ISI\})=\frac{\prod_{i}p\left({ISI}_{i}|\text{stimulus}\right)p(\text{stimulus})}{\sum_{\text{stimulus}}\prod_{i}p\left({ISI}_{i}|\text{stimulus}\right)p(\text{stimulus})}\;p(\text{choice}|\{\text{ISI}\})=\frac{\prod_{i}p\left({ISI}_{i}|\text{choice}\right)p(\text{choice})}{\sum_{\text{choice}}\prod_{i}p\left({ISI}_{i}|\text{choice}\right)p(\text{choice})}$$

### Synthetic controls

To test whether the ISI-based single-trial Bayesian decoder performance was indistinguishable from chance, synthetic spike trains were constructed for each trial of a cell by randomly sampling with replacement from the set of all observed ISIs under any task condition. We then trained and tested the decoding algorithm as described above (‘Single-trial, single-cell, ISI-based Bayesian decoding’) on these synthetic datasets. If a cell’s decoding performance was not statistically different from that of the synthetic dataset then that cell was excluded from all analyses involving decoding performance. For both true data and synthetic controls, datasets were split randomly into 10 folds and cross-validated 10 times, once to test accuracy on each fold. This process was performed 500 times to produce a total of 5,000 sample observations from true and control datasets. Significance from the null was assessed by comparing the distributions of observed sample observations from true and control data using a Mann-Whitney U test.

### Task-encoder threshold calculation

Cells were classified as significant task encoders using a decoding performance threshold that was fitted based on observed variance around chance. Decoding performance below chance was used as an estimate of decoding performance variability. Accordingly, the gap between the 5^th^ percentile and chance was taken as the threshold above chance for designating a cell as a “task-encoder”. In our dataset, this amounts to a threshold of 56% for stimulus decoding accuracy and 59% for choice decoding.

### Single-trial, ISI-based Bayesian ensemble decoding

To decode from neural ensembles, we generalized the approach for the single-unit case. The ISI probability distributions for each task condition for each neuron were calculated as in the single unit case. When decoding the spike trains from multiple units, each unit’s likelihood function independently updates the posterior probability of the stimulus (or choice). This is equivalent to the assumption that the ISIs observed from each neuron are independent.

(6) $$p\left(\text{stimulus}|\left\{{\text{ISI}}_{\text{ensemble}}\right\}\right)\;=\frac{\prod_{n}^{\text{ensemble}}\prod_{i}p\left({ISI}_{i}|\text{stimulus,}\;n\right)p(\text{stimulus,}\;n)}{\sum_{\text{stimulus}}\prod_{m}^{\text{ensemble}}\prod_{i}p\left({ISI}_{i}|\text{stimulus,}\;n\right)p(\text{stimulus,}\;n)}\;p\left(\text{choice}|\left\{{ISI}_{\text{ensemble}}\right\}\right)\;=\frac{\prod_{n}^{\text{ensemble}}\prod_{i}p\left({ISI}_{i}|\text{choice,}\;n\right)p(\text{choice,}\;n)}{\sum_{\text{choice}}\prod_{n}^{\text{ensemble}}\prod_{i}p\left({ISI}_{i}|\text{choice,}\;n\right)p(\text{choice,}\;n)}$$

Due to the fact that the number of possible neural ensembles increases combinatorically with increasing ensemble size, it was computationally intractable to perform analysis on all possible ensembles. For this reason, it was necessary to draw smaller samples of ensembles to analyze. To sample an ensemble of size n_1_, we randomly selected n_1_ neurons from the same recording without replacement. Because the total number of possible ensembles of size n_1_ rises exponentially with the number of neurons recorded in a session, n_2_, randomly drawing samples from the entire recorded population would bias results toward overrepresenting ensembles from high-yield recordings. In order to prevent this, we limited the number of size n_1_ samples drawn from each recording to be equal to n_2_. In this way, the number of ensembles of size n_1_ from each session scales linearly with the number of cells from each recording, n_2_, and every neuron recorded is equally represented in sampled ensembles.

### Optogenetic inactivation

For optogenetic inactivation of M2→ACtx projection neurons, retrograde AAVrg-CKIIa-stGtACR2-FusionRed (Addgene) was injected bilaterally into the auditory cortex at least two weeks prior to any optogenetic experiments (480 nL per hemisphere, 20 nL/min injection speed). The day prior to the start of optogenetic experiments we implanted bilateral cranial windows over M2 for optical illumination. Light was delivered to the surface of the brain with an optic fiber (200 μm diameter, ThorLabs) coupled to a 473nm diode laser (LuxX+, Omicron). Blue light pulses were triggered at tone onset and lasted 100ms (473nm wavelength, 2.5 mW mm^−2^). The laser was on for 50% of the trials, which were randomly interleaved with laser-off trials. Animals underwent photoinhibition for 12 days during post-reversal training corresponding to one standard deviation above the average number of days control animals take to learn the post-reversal stage (average plus standard deviation: 9.75± 2.60 days). The duration of photoinhibition was divided into 3 phases corresponding to the 3 phases of post-reversal learning. Opto phase 1 corresponds to the first 2 days of photo-inhibition; opto phase 2 corresponds to days 3–4; and opto phase 3 corresponds to days 5–7. There were two sham conditions: (1) stGtACR2-expressing excitatory neurons with no optical stimulation (AAVrg-CKIIa-stGtACR2-FusionRed; Addgene; n=3) and (2) mCherry-expressing excitatory neurons with optical stimulation (AAVrg-CKIIa-mCherry; Addgene; 473nm wavelength, 2.5 mW mm^−2^; n=1).

Optogenetic inactivation of M2 neurons was confirmed using in vivo electrophysiological recordings in M2. Retrograde AAVrg-CKIIa-stGtACR2-FusionRed (Addgene) was injected bilaterally into the auditory cortex (480 nL per hemisphere, 20 nL/min injection speed). Following 3 weeks to allow for viral expression, animals were head-fixed as described above (‘In vivo electrophysiology during behavior and passive exposure’) and a craniotomy was made over M2 (+2.34 mm AP, 1.4 mm ML from Bregma). A 64-channel silicon probe (Cambridge Neurotech) was inserted into M2 and blue light pulses were delivered with an optic fiber placed above the surface of M2 (473nm wavelength, 2.5 mW mm^−2^, 100 ms pulse duration).

Viral expression was confirmed using immunohistochemistry. In brief, mice were perfused with 4% paraformaldehyde, brains were removed and post-fixed in 4% paraformaldehyde for 24h at 4 °C, followed by immersion in 30% sucrose for 24–48 h at 4 °C. Brains were embedded in Optimal Cutting Temperature compound and stored at −20 °C before sectioning. Slices 40-μm thick were cut using a cryostat and stained and mounted using standard immunohistochemistry histological methods. ProLong Diamond Antifade Mountant with DAPI (4′,6-diamidino-2-phenylindole, Thermo Fisher Scientific).

### Movement tracking during behavior

Video recordings of behaving animals were synchronized with neural recordings. Animal movements during behavior were monitored with a camera (The Imaging Source, DMK 23U618) fitted with a zoom lens (Thorlabs, MVL16M23) and illuminated with infrared light (830 nm, Mightex SLS-0208-A). Video recordings and timestamps for each frame were recorded (frame rate: 100 frames/s) using Camera Control software^39^. We used a markerless pose estimation method (DeepLabCut^32^) to identify and track orofacial movements including the mouth, nose, and whisker. DeepLabCut was first trained with manually labeled frames selected from 5 videos from several behaving mice and then automatically evaluated for model accuracy. Post-processing included kinematic analysis of animal movements and the speed of animal movements for each video frame was calculated (DLC2Kinematics module).

To identify movement onset, for each frame, we calculated the difference between the average speed in 100 frames onward and the average speed in 100 frames backward. Movement onset was determined as the time of the first frame within 100 frames at which the calculated speed difference exceeds 2 standard deviations from the mean. A 0.5-second window starting from movement onset was defined as “After”, and a 0.5-second window prior to movement onset was defined as “Before”. We then calculated the average speed within these windows for each body part of interest (Extended Data Fig. 5).

The trial-averaged firing rate of both classically and non-classically responsive cells were calculated and aligned with the movement onset in a 1-second window, which includes the before and after movement periods.

### Dimensionality Analysis

We used the “participation ratio” definition of dimensionality^37,38^ which is typically written

(7) $$D=\frac{{\left(Tr\;C\right)}^{2}}{Tr\;C^{2}}$$

where C is the covariance matrix for the activity of n neurons defined as

(8) $$C=\left[\begin{array}{ccc} {Var}_{i} & \cdots & {Cov}_{in}\\ \vdots & \ddots & \vdots \\ {Cov}_{nj} & \cdots & {Var}_{n} \end{array}\right]$$

and ${Var}_{i}$ is the variance of the activity of unit i and ${Cov}_{ij}$ is the covariance in the activity between units i and j. Expanding the numerator and denominator of the “participation ratio” in terms of variance and covariance we find,

(9) $${\left(\text{Tr}\;C\right)}^{2}=\sum_{i=1}^{n}{\text{Var}}_{i}^{2}+\sum_{i\neq j}{\text{Var}}_{i}{\text{Var}}_{j}\;\;=n\langle {\text{Var}}_{i}^{2}\rangle +n\left(n-1\right)\langle {\text{Var}}_{i}{\text{Var}}_{j}\rangle$$

and

(10) $$\text{Tr}\;C^{2}=\sum_{i=1}^{n}{\text{Var}}_{i}^{2}+\sum_{i\neq j}{\text{Cov}}_{ij}^{2}\;\;=n\langle {\text{Var}}_{i}^{2}\rangle +n\left(n-1\right)\langle {\text{Cov}}_{ij}^{2}\rangle$$

where 〈·〉 indicates the average over the entire population of n neurons. This results in a simplified form for the dimensionality in terms of the single-unit variances and pairwise covariances

(11) $$D=\frac{1\;+\;(n\;-\;1)\frac{\left\langle {Var}_{i}{Var}_{j}\right\rangle }{\left\langle {Var}_{j}^{2}\right\rangle }}{1\;+\;(n\;-\;1)\frac{\left\langle {Cov}_{ji}^{2}\right\rangle }{\left\langle {Var}_{i}^{2}\right\rangle }}$$

In the large n limit this expression simplifies to

(12) $$D_{\infty }=\underset{n\to \infty }{lim}\;D=\frac{\left\langle {Var}_{i}{Var}_{j}\right\rangle }{\left\langle {Cov}_{ij}^{2}\right\rangle }$$

The numerator and denominator of this expression can be estimated directly from the data in each recording session in each learning phase. To estimate the variability in the numerator, we bootstrapped our calculation of dimensionality by sampling with replacement N_1_ neurons per animal per phase where N_1_ is the total number of neurons recorded per animal per phase. Because covariance is a pairwise measure and estimates of covariance can only be made between neurons recorded in the same session, we assume that our major source of variability for the denominator comes from variability between groups of neurons and we instead sample with replacement N_2_ recording sessions per phase where N_2_ is the total number of sessions recorded per phase to estimate the variability in the denominator. We then calculate the variance and covariances of all neurons from each sampled session, and calculate the dimensionality as described above. 2,000 bootstrap samples of dimensionality were made, and the mean and s.e.m. were calculated from these estimates.

### Statistical Analysis

All statistical analyses were performed in Python. One or two-tailed independent and relative t-tests, Mann-Whitney U tests, and Wilcoxon signed rank tests were performed appropriately based on hypothesis testing. Tests are selected based on whether data are paired or unpaired and on data normality according to the D’Agostino-Pearson test. Spearman’s ranked correlation tests were used to test for correlations between variables. All tests were multiple-comparison corrected with Benjamini-Hochberg corrections when appropriate. Error bars and shading on line plots indicate ± s.e.m. unless otherwise indicated. *p<0.05, **p<0.01, ***p<0.001, ****p<0.0001. For statistics of the population as a whole (percent non-classically responsive, percent best-frequency tuning), we used bootstrapping to estimate mean and s.e.m. 2,000 estimates of the statistic were calculated by sampling with replacement n neurons from each recorded animal, where n is the total number of neurons recorded for that animal; for the estimate of dimensionality, we sampled with replacement N sessions from each phase where N is the total number of recorded sessions per phase. To test for differences, a permutation test with 250,000 sample observations was used to calculate the p-value for a minimum detectable p-value of 4×10^−6^.

## Data Availability

The data that support the findings of this study are available on Github (https://github.com/InsanallyLab/TothSidleck2024) and from the corresponding authors upon reasonable request.
